# The Treatment and Filling of Pulpless Teeth as Learned from Papers Read at the World's Dental Congress, A. D., 1893

**Published:** 1894-12

**Authors:** S. D. Banes

**Affiliations:** CHICAGO, ILL.


ARTICLE IV.

### THE TREATMENT AND FILLING OF PULPLESS TEETH AS LEARNED FROM PAPERS READ AT THE WORLD'S DENTAL CONGRESS, A. D., 1893.

BY S. D. BANES, D. D. S., CHICAGO, ILL.

Read before the Odontographic Society, May, 1894.

*Mr. President and Gentlemen:*—The object of this paper is to bring before this society for discussion the methods of treating and filling pulpless teeth, which were presented in papers or exhibited in clinics at the World's Dental Congress, of 1893.

Upon the second day of the session Dr. J. H. Woolley prepared and filled the roots of a superior second molar for a crown. The tooth was decayed very high up, so that the pulp chamber was fully open and all the canals accessible. Dr. Woolley cleaned out the canals with his broaches. He then volatilized alcohol in them by means of a Small's canal dryer. He then thoroughly dried the tooth with Richmond's hot air dryer, and tested the dryness of the canals by inserting the probe of a root canal dryer of his own design, with which you are probably familiar. Next he pumped eucalyptol into the canal, and volatilized it with hot air, so as to drive it to the end of the tubuli, or fill them with vapor. Finally he flooded the canals with eucalyptol, the pulp chamber with chloroform, and inserted some cones. After driving off the chloroform by hot air he dismissed the patient.

The trouble with that kind of a filling is that there is too much liquid used. I question whether the eucalyptol passed to the apical foramen, and I do not believe that the gutta-percha, upon which we must depend to stop those passages permanently, passed so far. When the cone reached the small portion of the canal it would act as a piston and drive the liquid through the foramen, producing pain, the operator concluding thereform that his filling was complete; but we think in time, and in a short time at that, the eucalyptol will diffuse away, leaving the apical portion of the canal above the cone unfilled.

On the third day Dr. W. H. Richards exhibited his method of filling tortuous canals with a metallic fiber. He cleaned out the canal and moistened the walls with oil of cassia, pumping it in with a broach. He picked up the metallic fiber on the moistened broach and passed it into the canal, working it up until by the sense of touch he determined that it had reached the apical foramen.

This method seems to me to be open to many and very serious objections. First, I do not believe any man can be sure when he has reached the apex with his metallic fiber. In ninety-nine cases he will force it through the foramen, or stop before reaching it, to one where the fiber would just pass to the foramen and stop there. Second, the fiber does not fill the canal, and the tooth is left only a little better off than it would be if the canal were entirely empty. Third, if you can get a probe, into a canal you can file a gold wire as fine or finer than the probe and after pumping in the chloroform you can drive in your gold wire. The chloro-percha will fill in around the wire and you thus have a more perfect filling than is afforded by a loose metallic fiber in the canal. Still further, if you can pass a probe part way into a canal, with patient effort you can fill that canal to the end with solid Hill's stopping.

In the discussion of the question "Can apical pericementitis result about a root that has been thoroughly sterilized and filled?" some valuable points were brought out. Dr. Fernandez said that he hoped the time would come when arsenic would no longer be used to devitalize the pulps of teeth. He dwelt upon the fact that we are using a very strong and uncontrollable poison, that we do not know how far its energy extends, or exactly how it acts in devitalizing the organ. He advocated some mechanical method, giving gas, if necessary, to avoid the pain. If obliged to use arsenic he would use only enough to devitalize the body of the pulp and desiccate the rest with tannin and glycerine or tannic acid in glycerol. If he could not then remove all of the tissue he would digest it in pepsin and wash it out. Dr. Fernandez said he thought a large number of cases of pericementitis were caused by the action of the arsenic or the poisonous medicaments used in the destruction of pulps.

Dr. I. P. Wilson pointed out the way in which the arsenic probably caused the pericementitis. If you will notice a longitudinal section of a tooth you will see a small portion of the apex of the root that is entirely composed of cementum; this small portion of cementum is undoubtedly nourished by blood vessels from the pulp, and if the pulp is devitalized by the arsenic beyond this point its nourishment is cut off. It then acts as a source of irritation, causing that permanent uneasy feeling at the end of the tooth. That portion of the tooth is prone to become necrosed after the death of the pulp, as is shown by the number of cases of chronic abscesses, which cure up nicely after drilling in and cutting off the end of the root.

During the same discussion, Dr. A. O. Rawls, of Lexington, Ky., said that to protect the teeth perfectly it was necessary to fill the canals to the end, and also the tubuli of the dentine to their extremities. In order to do this he advocated drying the entire tooth from crown to apex with hot air. Then applying some ethereal solution as a varnish, that will fly like a flash to the farthest extremity of the tubuli.

I would want to try a good many experiments with teeth in plaster and wet plaster, so that I could test the methods by microscopical examination, before adopting the method in the mouth.

On the fourth day of the session, the paper of Dr. Poinsot, of Paris, France, upon the subject of the "Extraction of the pulps of teeth in a calcified state of trepanning," was read by the chairman of Section IV. The Doctor claims that physiology teaches us that the teeth receive their nourishment from two different sources, viz: the external and the internal; the external life of the teeth through the peridental ligament, and internal through the pulp, the function of which consists in securing the calcification of the organ in the internal part of the periphery, diminishing the pulp chamber in a progressive way. Calcification is generally complete at the age of thirty-five or forty years, and no matter what the cause may be, either local or general; when once calcification is complete he considers it advantageous to perform the operation of trepanning, which he accomplishes by drilling through the axis of the central pulp chamber, using a small drill for single rooted teeth, and a large drill for teeth with more than one root, carefully removing the enamel at the spot where the drill acts, by the use of drills made from small diamond chips. The way to extract the dental pulp with muscular fillet attached is by the use of a nerve broach, which is used after obtunding the tissues with cocaine phenate, or if coot canal is mechanically inaccessible, by the use of electro cautery. Finally, by filling the canals and remainder of cavity in the usual way. He suggests two ways of indicating when calcification is complete. First, when the gum following the peridental ligament surrounds and enclasps the crown of the tooth. And second, by a careful examination by means of an electric light.

The Doctor's patients have the sympathy of the author of this paper at least, if not of my auditors, inasmuch as his ideas as set forth in the paper presented, necessitates the wholesale slaughter of pulps, without any exception, as soon as the calcification is complete—which is claimed to be from thirty-five to forty years—iu order to preserve their usefulness and longevity. Such a method of treatment may possibly be excusable in Paris, but for the sake of humanity keep the Doctor and his trepanning on the other side of the water.

The first of the two papers on the treatment of root canals was that sent by Dr. Miller, of Berlin, Germany. It was read in the general session of the third day. The paper reports his experiments with methods to avoid the removal of dead pulps from the canals of bicuspids and molars. The method is one of mummification. The object sought is to find some substance with which to impregnate the remaining portion of the pulp, so that it will not decompose in the future. He points out seven characteristics or qualities, which are briefly:

1. It must be a strong antiseptic.
2. It must be soluble and diffusible.
3. It must not be too soluble and diffusible.
4. Coagulating action is desirable.
5. It must not be irritating to the pericementum.
6. It must not discolor the teeth.
7. Solids are better than liquids.

The only substance that I know of that meets all these conditions is gutta-percha in a clean, aseptic canal.

The first method used by Dr. Miller was to remove the body of the pulp after completely devitalizing it by the application of a small tablet containing

| | | |
|---|---|---|
| Sublimate (Hy. Co.) | - | 0.01 gram. |
| Baracic Acid, | - - - | 0.02 " |

Or,

| | | |
|---|---|---|
| Sublimate, | - - - | 0.01 gram. |
| Sodium Chloride, | - - | 0.02 " |

The tablets were applied, crushed slightly and moistened, then covered with a layer of gold foil and the cavity filled. But in about 30 per cent. of the cases this method caused violent pain because of the rapid diffusion. The painfulness resulting from the use of the first compound led to the use of

| | | |
|---|---|---|
| Sublimate, | - - - | 0.0075 gram. |
| Thymol, | - - - | 0.0075 " |

The thymol was used to prevent the too rapid diffusion of the sublimate. He also used

| | | |
|---|---|---|
| Sublimate, | - - - | 0.005 gram. |
| Thymol, | - - - | 0.005 " |
| Tannin, | - - - | 0.005 " |

But the tannin discolors the tooth. He also used the cyanide and the salicylate of the mercury, but expressed himself as having achieved the best results from using diaphtherin (oxychinaseptol), a new antiseptic recently introduced by Emmerich. The Doctor reports that in over two hundred cases at the University of Berlin, where this method had been used, only one failure has come to his knowledge, which we are constrained to admit is a good record if he, in fact, heard of all that failed.

As Dr. Miller observes, such a method can only be fairly tested by time, and in any case should be used very seldom, and only in teeth that would otherwise be treated by the forceps.

I think we had all looked for a valuable paper from Dr. Miller, but I must confess I was very much disappointed in it. I think that as with the mastery of geometrical science, no royal road to an easy solution of the most difficult problems has yet been blazed out for the student's guidance, so with root filling in dentistry, no absolutely perfect and easy method capable of universal application has yet been discovered.

And as Dr. Miller admits the present method of filling the anterior teeth is not likely to be improved upon, and can be trusted to give success in a respectable number of cases, is it not fair then to say that the greater number of failures in filling the molars and bicuspids are due to the carelessness or impatience of the operator? In my opinion two things are absolutely necessary in the treatment of a root canal. First, it must be aseptic, Second, it must be sealed at the apex and the pulp chamber. I believe it would make little difference if the canals were absolutely empty if the apical foramen and the crown cavity were positively sealed: But to seal the foramen all the rest of the canal must be filled. Dr. Miller's method does not provide the second condition, and so long as the apical foramina are open so as to make the canals receptacles for serum, trouble is sure to follow sooner or later. I think for myself I would sooner extract a tooth than to make such an operation simply to save time and expense. If the tooth could be temporarily saved by this method it would surely be saved longer by a careful filling.

In the discussion of this paper Dr. Frank Abbott, of New York, brought out his method of filling root canals. In the first place, he said that he very seldom used arsenic to devitalize a tooth pulp, and when he did so it was only to relieve pain.

When a tooth comes to him for treatment, with the remains of a dead pulp in it, he opens into the pulp chamber so that he can have easy access to the canals. He then uses a one in ten thousand solution of the bichloride of mercury (one grain to twenty ounces), syringing out all the canals as thoroughly as possible. With a broach he probes into the canals so as to stir up the contents, and syringes again, repeating the process until the canals are clean, and the solution in coming out will not stain a white napkin. When he has thoroughly washed it out he fills it with oxychloride of zinc, to which he adds one drop of a one in two thousand bichloride solution. He said this is the material that mummifies or holds the substance which remains in the root of the tooth in such a condition as to give no trouble. He cleans out the canals and fills at the same sitting, before dismissing the patient, painting over the gum with aconite and iodine, as a counter-irritant.

The paper on this subject which has created the greatest amount of interest was that presented by Dr. Emil Schreier, of Vienna. He has given us a positively new method for removing dead and putrescent pulps, and all operators are interested in it because the value of such a new method can only be tested by a large amount of clinical experience.

I presume you are all familiar with his method. He prepares a mixture of metallic sodium and potassium (about two parts sodium and one part potassium) of such a consistency that when a platinum broach is plunged into it, through the parafine coating, a film of the alloy will adhere and be carried on the broach, and the broach thus laden is passed into the moist canal. The metal decomposes the watery contents liberating the hydrogen and forming the hydroides of the metals (caustic potash and caustic soda), which in the nascent state actively decompose the organic matter of the pulp, saponifying it (if that is a proper term to apply, as there is little or no fat in the tissue.) At any rate, the tissue is actively decomposed and rendered soluble, so that it is readily washed out with water.

The substance destroys the germs present partly by the heat evolved and partly by the chemical products. Of course this method is only applied with the rubber dam in position to protect adjacent tissues.

The questions which arise in connection with this method, some of which were suggested by Dr. Schreier, are;

1. Is there danger of forcing septic matter through the apical foramen, when the broach is first plunged into the canal?

2. Is the heat evolved too great?

3. Is there danger of an explosion?

4. Is there danger that the substances formed will pass through the foramen and set up irritation of the peridental membrane?

The latter is far the greatest danger. I would be very much afraid that in case ot an open foramen, some of the caustic substance might get into the apical space, and if it should, trouble would doubtless manifest itself.

In connection with Dr. Schreier's paper, I have consulted some fifteen dentists in good practice regarding the Doctor's method, and found only two of that number who have ventured its use since Congress adjourned, and one of the gentlemen was very much gratified with the results, while the other's experience was quite the reserve.

I have since been advised that other gentlemen present have used this method in their practice before the meeting of the World's Dental Congress last year, and some of them with much satisfaction, and we should certainly be pleased to hear from any such parties.

Through the courtesy of Mr. Fred. Noyes, I am enabled in this connection to submit the following letter from Prof. Noyes, of the Rose Polytechnic Institute, of Terre Haute, Ind., a prominent chemist in our sister State, regarding the chemical action of the compound used by Dr. Schreier. Prof. Noyes' letter is as follows:

"I do not see how anything could be gained by the use of metallic sodium and potassium in the manner you indicate, which would not be gained equally as well by the use of sodium hydroxide, or potassium hydroxide, and with far less danger. The metals I should think likely to lead to ugly accidents in such a use. The hydroxides would, I think, have little effect on bony tissue, fats would be saponified, and other organic matter in general would be disintegrated by them."

We had hoped to have access to the official publication of the entire transactions of the Congress, but owing to delays of various kinds it has not yet been published.—*Dental Review.*

